# *Cellular and Molecular Biology Letters* goes open access with BioMed Central

**DOI:** 10.1186/s11658-016-0001-5

**Published:** 2016-07-28

**Authors:** Aleksander F. Sikorski

**Affiliations:** Department of Cytobiochemistry, Faculty of Biotechnology, University of Wrocław ul. F. Joliot-Curie 14a, 50383 Wrocław, Poland

It is my great pleasure to announce the launch of a new alliance between our journal, *Cellular and Molecular Biology Letters (CMBL)*, and an international publisher, BioMed Central (part of Springer Nature). This event opens the 21st year of publication of our journal and changes the model of publication to an open system. Therefore, I would like to take this occasion to thank my colleagues, Professors Jan Szopa and Arkadiusz Kozubek* and Ms Małgorzata Nietubyć for a wonderful 20 years of a lasting and common effort to publish the journal. I remember, in particular, the pioneering years, when we started the journal without financial support and with rather weak institutional help when conditions were very challenging. Without their help, support and contribution throughout this formative period, this venture would not have been possible.

I would like to thank all of our authors and readers who supported our initiative since its start in 1996. Of great importance was the tireless effort of the reviewers in taking care of the standards of the publications. Their contribution has been invaluable. The contributions of the first Editorial Board Members who helped us a lot through their advice, in particular our colleagues, Professors Stanisław Przestalski, Tadeusz Chojnacki and Włodzimierz Korohoda, have been greatly appreciated.

Our journal is published by the University of Wrocław, in collaboration with the Polish Society for Cell Biology, along with more than 15 years of lasting support from the Polish Ministry of Scientific Research and Higher Education. I would like to take this occasion to express our gratitude to these institutions.

Of course, the greatest support that we received was from all of our contributors who submitted work of such a standard that we were able to enter world-wide databases, in particular, the ISI Web of Science, so quickly. The quality of the contributions is reflected in the recently published Impact Factor for the journal, which is 1.753 for the past year, along with a five-year Impact Factor of 1.681.

*CMBL* is a forum for researchers, covering the broad areas of cell and molecular biology, including: the cell cycle and its regulation, cell differentiation, cell traffic and signaling, stem cells, gene expression regulation, cellular membranes, cytoskeleton and molecular biotechnology, not excluding other aspects of studies such as cellular proteomics, genomics and advanced imaging technologies. Research, review, short report and mini-review articles are all welcome.

Although our journal has developed well and to the point that it now has a small, but stable, circle of authors and readers, we have considered now for quite some time on how to get into an open access model of publication. Now, through establishing the collaboration with BioMed Central, the world-famous open access publisher, we gain this opportunity. Therefore, we hope that, with this new arrangement, the journal will continue to grow in its readership as well as upon its impact within the scientific community that it is trying to reach. Fortunately, the open access journals have become a standard vehicle of publication of the newest achievements of the research in cell and molecular biology. Both the Editorial Team and I, believe that the choice we have made are vital for the future of the journal and that we will be successful in achieving our goal. However, we still need the support of all of you, both our authors and our readers, in this.

With this move towards open access comes also a change in the way that the journal will charge for its services, with the introduction of a single flat-fee as an article processing charge (APC) for successful manuscripts. This is an inevitable part of the process of open access publishing, where the costs associated with an open access format are covered by the APC. We believe that, overall, this charge structure represents a cost-effective publishing route, especially when the wider reach of the open access format will bring manuscripts to the attention of a much larger audience, with a corresponding increase in weighting in the impact factor. On behalf of the Editorial team, I promise that our priority is the quality of research presented by the manuscripts it publishes, its novelty, technical advance and importance in learning molecular mechanisms underlying normal and pathological cell biology. All manuscripts will be treated equally, honestly peer-reviewed and published as soon as they will be accepted. So, we invite you all, as authors, to publish with us.

We trust that our authors will be satisfied with the publication service they receive from our Editorial Office and BioMed Central. It is my strong belief that this alliance will secure the stable progress of the journal and help it make a greater impact in the field of the molecular biology of the cell. I also hope very much that it will continue to provide a friendly vehicle for the publication of important and significant achievements in global research towards progress in learning about how cells function.

Editor-in-Chief, *Cellular and Molecular Biology Letters*

*Sadly, passed away on May 20, 2016

